# Comparison of homoeolocus organisation in paired BAC clones from white clover (*Trifolium repens *L.) and microcolinearity with model legume species

**DOI:** 10.1186/1471-2229-10-94

**Published:** 2010-05-24

**Authors:** Melanie L Hand, Noel OI Cogan, Timothy I Sawbridge, German C Spangenberg, John W Forster

**Affiliations:** 1 Department of Primary Industries, Biosciences Research Division, Victorian AgriBiosciences Centre, 1 Park Drive, La Trobe University Research and Development Park, Bundoora, Victoria 3083, Australia; 2 Molecular Plant Breeding Cooperative Research Centre, Australia; 3 La Trobe University, Bundoora, Victoria 3086, Australia

## Abstract

**Background:**

White clover (*Trifolium repens *L.) is an outbreeding allotetraploid species and an important forage legume in temperate grassland agriculture. Comparison of sub-genome architecture and study of nucleotide sequence diversity within allopolyploids provides insight into evolutionary divergence mechanisms, and is also necessary for the development of whole-genome sequencing strategies. This study aimed to evaluate the degree of divergence between the O and P' sub-genomes of white clover through sequencing of BAC clones containing paired homoeoloci. The microsyntenic relationships between the genomes of white clover and the model legumes *Lotus japonicus *and *Medicago truncatula *as well as *Arabidopsis thaliana *were also characterised.

**Results:**

A total of four paired homoeologous BACs were selected and sequenced to generate 173 kb of overlapping sequence between the O and P' sub-genomes. Equivalent gene content was generally observed, apart from small-scale deletions, in contrast to conservation of intergenic sequences, which varied between the four selected regions. Measurement of the number of synonymous substitutions between homoeologous genes led to estimation of a 4.2 million year divergence time between the two sub-genomes. Microsynteny was observed between the genomes of white clover and *L. japonicus *for all four targeted regions, but corresponding *M. truncatula *genomic regions were only identified for two BAC pairs.

**Conclusions:**

This study describes the first analysis of sub-genome structural conservation across selected genomic regions in white clover. Although the high levels of sequence conservation between the O and P' sub-genomes would complicate efforts for whole genome sequence assembly, the conserved microsynteny with model legume genomes, especially that of *L. japonicus*, will be highly valuable for the future of white clover genomics and molecular breeding.

## Background

Allopolyploid genome constitutions, arising from hybridisation between related diploid taxa, are ubiquitous features of plant genetic architecture [1,2]. Examples of agronomically important allopolyploid species include bread wheat (*Triticum aestivum *L.: 6x), cotton (*Gossypium *species: 4x), canola (*Brassica napus *L.: 4x), oat (*Avena sativa *L.: 6x), strawberry (*Fragaria *× *ananassa *Duch.: 8x), peanut (*Arachis hypogaea *L.: 4x) and white clover (*Trifolium repens *L.: 4x). The majority of allopolyploid taxa behave as functional diploids due to the action of genes controlling pairing between homoeologous chromosomes, such as the *Ph*1 locus of allohexaploid bread wheat [3,4]. The degree of divergence between sub-genomes in terms of evolutionary history or sequence diversity may be variable. Some allopolyploid taxa (such as cotton: [5]) are relatively ancient in evolutionary terms, while others have arisen recently (such as allohexaploid wheat within human pre-history: [6]).

As high-throughput sequencing technologies rapidly improve, it is becoming more realistic to produce draft genome sequence assemblies of important crop species. However, this process is highly complicated by polyploidy, especially when sub-genomes are closely related, which might be expected to dispose towards chimaeric contig assembly. One strategy to overcome this problem is to first sequence the relevant genomes of diploid progenitor taxa, when such information is available. An understanding of relatedness between the sub-genomes is hence essential prior to any such genome sequence assembly efforts. Studies of homoeologous sub-genome conservation within various paleopolyploids have revealed both low (maize [7]) and high (soybean [8]) levels of gene retention, while true polyploids such as cotton contain both conserved genic and intergenic regions [9,10].

White clover is an outbreeding allotetraploid (2n = 4x = 32) species, and an important forage legume in temperate grassland agriculture. The evolutionary history of white clover has been a matter of considerable controversy, but recent molecular phylogenetic studies [11] have implicated *T. occidentale *D.E. Coombe (Western clover) and *T. pallescens *Schreber as putative diploid progenitors. Cloning and sequencing of PCR amplicons from protein-coding genes derived from white clover and the diploid taxa has permitted resolution of homologous, homoeologous and paralogous sequence variation categories, and revealed high levels of sequence similarity (c. 95%) between the two sub-genomes. One of the white clover sub-genomes is closely related to that of contemporary *T. occidentale*, and has been designated O, while the other sub-genome is more distantly related to both *T. occidentale *and *T. pallescens*, and has been designated P' [12]. Genome-specific sequence haplotypes have been defined based on the presence of homoeologous sequence variants (HSVs) between homoeoloci. The detection of genome-specific sequence features allows the selection of bacterial artificial chromosome (BAC) clones containing paired homoeoloci. Subsequent characterisation provides an evaluation of microscale evolutionary divergence mechanisms between the two sub-genomes.

Sequencing of white clover BACs also permits analysis of microsynteny with the genomes of *Lotus japonicus *and *Medicago truncatula*, which were chosen as model legume species and for whole-genome sequencing projects due to modest genome size, diploid genetics and short life-cycles [13,14]. *L. japonicus *and *M. truncatula *are members of the Hologalegina division of the Fabaceae and are assigned to the Robinoid and Inverted Repeat Loss Clade (IRLC) respectively, which share strongly supported sister relationships [15]. Of the two model species, white clover is more closely related to *M. truncatula*, as both are placed in the IRLC and are members of the Trifolieae tribe. Genomic resources available from model legumes are hence potentially extremely valuable for the development of genetic tools in white clover. An evaluation of microsynteny will consequently provide a measurement of the value to be gained from each of the model species.

This study describes the analysis of four paired white clover homoeologous BACs and provides the first evaluation of divergence between the co-resident O and P' sub-genomes for both genic and intergenic regions. A primary estimation of the degree of evolutionary divergence for the two sub-genomes was also obtained. Finally, syntenic relationships between the genomes of white clover and the model legumes, as well as *A. thaliana*, were studied to reveal potential implications for translational genomics and whole-genome sequencing strategies.

## Methods

### BAC selection

Arrayed pools from a white clover BAC library constructed from multiple genotypes of cultivar Grasslands Huia [16] were screened by PCR for clones containing genes previously identified and characterised in white clover (*Krüppel*-like zinc finger protein - *ZPT2*, dehydration responsive element binding protein - *DREB3*, dehydrin - *DHNb *and anthocyanidin reductase - *ANR *[12,17]). Each PCR reaction had a total volume of 20 μl and contained 1 × Immolase PCR buffer, 1.5 mM MgCl_2_, 200 μM dNTPs, 0.25 μM each primer, 0.4 units Immolase DNA polymerase (Bioline) and approximately 20 ng BAC DNA template. Cycling conditions for amplification of all genes were 95°C for 15 minutes, 35 cycles of 95°C for 1 minute, 55°C for 1 minute, 72°C for 1 minute followed by a final extension of 72°C for 7 minutes. PCR products generated from single clones were sequenced to determine the sub-genomic origin of the clone. A total of 5 μl of each PCR product was purified through addition of 2.5 units of Exonuclease I and 2.5 units of shrimp alkaline phosphatase (SAP). The reaction was incubated at 37°C for 60 minutes and enzymes were inactivated at 80°C for 15 minutes. Each sequencing reaction contained 0.16 μM primer, 0.25 μl BigDye^® ^Terminator v3.1 (Applied Biosystems), 0.875 × BigDye Sequencing Buffer (Applied Biosystems) and 0.5 μl of purified PCR product in a final volume of 10 μl and subjected to cycling conditions as described in the BigDye v.3.1 protocol. The extension products were purified with ethanol, sodium acetate and EDTA following the BigDye^® ^Terminator v3.1 Cycle Sequencing Kit protocol (Applied Biosystems) and electrophoresis was performed on the ABI3730xl automated sequencer. Target regions from three of the genes (*ZPT2*, *DREB3 *and *DHNb*) had previously been isolated and sequence determined from both white clover sub-genomes as well as *Trifolium occidentale *[12]. For the *ANR *selected clone, the *ANR *region was resequenced from *T. occidentale*. Of the two putative progenitor genomes, only that of *T. occidentale *was resequenced, as *T. pallescens *was previously shown to be more divergent from both the O and P' sub-genomes, and hence uninformative with respect to sub-genome attribution [12]. Of the BAC clones identified, those containing the sequence with the highest nucleotide identity to *T. occidentale *were designated O, while their counterparts were designated P'. Validated single nucleotide polymorphisms (SNPs) were previously discovered and validated for each gene and used for assignation to homoeologous groups (HGs) [12,18,19].

### BAC isolation, sequencing and assembly

BAC DNA was isolated using the QIAGEN large construct kit and sheared using either a nebuliser (Invitrogen) for 35 seconds at 50 kPA for BAC clones wc11l07, wc113f04, wc88n22, wc99k01 and wc88b23 or the HydroShear^® ^(GeneMachines) for 20 cycles with a speed code of 13 for BAC clones wc38j22, wc32k23, wc36e03, wc87b16 and wc84n20. Sheared DNA was blunt-end repaired, dephosphorylated and sub-cloned into the pCR^®^4Blunt-TOPO^® ^vector using the TOPO^® ^Shotgun Subcloning Kit (Invitrogen). Plasmids were transformed into TOPO10 chemically competent *E. coli *and grown overnight at 37°C on LB plates containing 75 μg/ml carbenicillin and 40 μg/ml X-gal to facilitate blue/white colony selection. White colonies were picked and the recombinant plasmid was amplified using the TempliPhi DNA Sequencing Template Amplification Kit (GE Healthcare).

The amplified template was diluted with the addition of 30 μl of ddH_2_O, of which 3 μl was used as template for sequencing, as previously described, with T7 and T3 primers. Vector and poor quality sequence was trimmed using Sequencher 4.7 (Gene Codes) and contigs were assembled using CAP3 [20] set to default parameters. Contigs generated previously from partial sequencing of the P' sub-genome *ANR*-selected BAC [17] were also included in the assembly of this clone sequence. Gaps present in the sequence assembly were filled by fully sequencing bridging sub-clones. In those cases in which no clones spanned the gap, primers were designed from flanking regions to amplify the bridging sequence from the isolated clone. Each PCR reaction was performed with 20 ng of BAC DNA as template, and was purified and sequenced as described previously.

The derived DNA sequences from the selected BAC clones were deposited in GenBank (Accession numbers GU443959-GU443960).

### Sequence analysis

Gene prediction was performed using alignment against a white clover EST library [21] and *ab initio *methods. These methods used a combination of predictions generated from FGENESH with *Medicago truncatula *parameters [22], Genscan with *Arabidopsis thaliana *parameters and GeneMark with *Medicago truncatula *[23] parameters. Predicted genes were subjected to BLASTX queries against the GenBank non-redundant (nr) database to assign putative function. To identify any putative coding sequences that the gene prediction methods failed to detect, 500 bp fragments of BAC sequence were also used as input for BLASTX searches against the GenBank non-redundant database.

Predicted genes were then classified as homoeologous when they shared the same putative function. Percentage identity between homoeologous coding and protein sequence was calculated using the Smith-Waterman algorithm, with a gap opening penalty of 10 and gap extension penalty of 0.5 (EMBOSS). Homoeologous genes were aligned using ClustalX 2.0.12 [24] and synonymous and non-synonymous substitutions were assessed using the model of Goldman and Yang [25] as implemented by the CODEML application of the package PAML [26]. The predicted time of divergence was then estimated using a molecular clock estimator of 6.1 × 10^-9 ^substitutions per synonymous site per year[27-29]. Using LAGAN set to default parameters [30] global pairwise-alignment was performed for homoeologous BAC sequences. Subsequent percent identity between the sequences was visualised through a VISTA plot [31]. Repetitive regions were identified from the *Arabidopsis thaliana *reference collection of repeats within Repbase using CENSOR [32] and tandem repeats were discovered using Tandem Repeats Finder [33].

Predicted coding sequences were subjected to BLASTN searches against all predicted genes within the *Lotus japonicus *[34] and *Medicago truncatula *(release 3.0) [35] genomes and against all coding sequence within the *Arabidopsis thaliana *genome (TAIR9) [36]. Regions of the model genomes were deemed to be syntenous to the sequenced white clover regions when they contained 3 or more predicted genes with an e-value less than or equal to 0.01. Conservation of intergenic regions between white clover and the model genomes was investigated using CoGe [37].

## Results

### Sequence assembly and annotation

A total of 8 BACs representing 4 homoeologous regions were selected using the genes listed in Table 1. For those BACs not sequenced to completion, all analysis was performed using that contig which contained the selected gene (*ZPT2*, *DREB3*, *DHNb *or *ANR*). Gene prediction methods identified a total of 48 genes across all BAC clones (Additional file 1), 18 which were classified as homoeologous in nature. On average, when data from both sub-genomes was combined, one gene was predicted every 9,148 bp and the total proportion of clone sequence occupied by gene space was 29.90%. The gene incidence and gene space values were similar between sub-genomes for regions B, C and D, but the O sub-genome sequence from region A appeared to be almost twice as gene-rich as the P' sub-genome sequence. This disparity was resolved when only overlapping regions were compared; the O and P' sub-genomes then displayed gene incidence values of one gene every 6,375 and 7,286 bp, and the proportions of sequence occupied by gene space were 46.42% and 33.09%, respectively. The original observation was due to the very low gene content in the extra 66 kb that was sequenced from the P' sub-genome. Of the 48 total predicted unique genes, 15 (31%) are supported by the identification of matching white clover ESTs and six of these are present in both sub-genomes. Of these six homoeologous genes, ESTs were identified only from the O sub-genome for one gene, from the P' sub-genome for two genes, from both sub-genomes for one gene and the sub-genome origin of the EST could not be determined for the remaining two genes. Following analysis of all predicted genes from both sub-genomes, the average exon length was 348 bp and the average intron length was slightly smaller, at 314 bp.

**Table 1:** Characteristics of eight BACs sequenced from white clover, representing four homoeologous regions

Region	BAC identity	Gene used to identify	Sub-genome	**HG**^**a**^	Assembly phase	Length (kb)	**Overlap length (kb)**^**b**^	Gene incidence (bp)	**% BAC composed of gene space**^**c**^
A	wc38j22	*TrZPT2*	O	3	2	61	51	6100	45.20
A	wc11l07	*TrZPT2*	P'	3	3	127		11545	23.05
B	wc113f04	*TrDREB3*	O	4	2	73	51	12166	28.34
B	wc88n22	*TrDREB3*	P'	4	3	101		10100	28.05
C	wc99k01	*TrDHNb*	O	3	3	152	50	8941	29.80
C	wc32k23	*TrDHNb*	P'	3	2	50		10000	23.47
D	wc88b23	*TrANR*	O	4	1	25	21	8333	36.77
D	wc36e03	*TrANR*	P'	4	1	24		6000	24.55

**Average**								**9148**	**29.90**

^a. ^Homoeologous group

^b. ^Regions of homoeologous BAC overlap were determined based on alignment of the gene used to identify the BAC.

^c. ^Gene space is defined as all nucleotides between the start and stop codon of the open reading frame and including all exons and introns.

### Comparison of homoeologous regions

**Figure 1:**
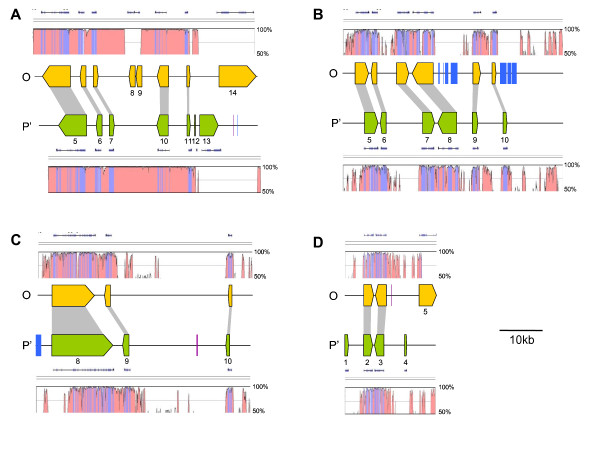
**Gene positions across four homoeologous regions of white clover**. Block arrows represent predicted genes, the arrow direction indicating the transcriptional orientation. Gene numbering corresponds to the nomenclature in Additional file 1, and homoeologues are linked by grey boxes. Coloured rectangles represent transposable elements: blue are Copia LTR retrotransposons and violet are DNA transposons. Plots above and below the gene schematic are VISTA plots representing percent nucleotide identity between the two sub-genomes. Lines above the plots indicate coding regions and blue shaded regions within the plot represent exons. The letters A, B, C and D correspond to the 4 sequenced regions, as described in Table 1.

Conservation of gene content, order and orientation between the O and P' sub-genomes were identified from examination of the overlapping sequence for each region (Figure 1). A total of 51 kb of sequence overlapped for regions A and B, 50 kb for region C and 21 kb for region D.

All of the 18 homoeologous genes that were identified across the four regions were conserved in order and orientation between the two sub-genomes. Exon/intron structure was also highly conserved, with all but two of the genes (B.6 and C.11) having the same number of exons and introns. The two exceptions to the rule both had an additional predicted exon in the O sub-genome gene copy. Of the 18 genes, those copies located in the P' sub-genome had, on average, larger exons with an average length of 360 bp, as compared to 318 bp for the O sub-genome copies. These differences are predominantly due to insertions or deletions within exons in either sub-genome rather than truncations or alternate start sites. The one exception to this is gene C.8 (MKRP2), which has an additional 141 bp 3' in the P' sub-genome copy. The average intron length was also slightly greater for the P' sub-genome (285 bp) as compared to the O sub-genome (268 bp). There was, however, no bias as to which sub-genome contained the larger introns for each pair of homoeologous genes. Of the 15 pairs of genes with an equal number of introns, six had larger introns in the O sub-genome, six in the P' sub-genome and three are of equal length.

The level of nucleotide identity between each homoeologous exon ranges from 85.9% to 100%, with an average value of 97.18% (Table 2). The level of conservation between introns was on average lower, as expected, but still very similar ranging from 66.4% to 100% nucleotide identity with an average of 88.93%. Nucleotide identity was calculated for each individual exon and intron pair to determine whether any consistent trend was present in either direction across the gene space, but no such trend was observed.

**Table 2:** Measures of identity and similarity between homoeologous genes identified across four regions of the white clover genome

Gene	Putative function	Average exon nucleotide identity (%)	Average intron nucleotide identity (%)	Protein sequence identity (%)	Protein similarity (%)	Ks	Ka
A.5	Predicted protein 2	99.1	99.8	98.8	99	0.0019	0.0026
A.6	Predicted protein 3	99.1	100	98.3	99	0.0165	0.0074
A.7	Galactose oxidase	99.9	98.6	99.7	99.7	0	0.0011
A.10	Adenine phosphoribosyltransferase	100	99.8	100	100	0	0
A.11	ZPT2	97.1	-	97.9	98.4	0.0409	0.0022
B.5	Bristled 1	85.9	88.4	86.3	87.5	0.0797	0.0059
B.6	Ethylene insensitive 3	94.2^a^					
B.7	bZIP transcription factor	97.1	62.6	97.1	98.2	0.0715	0.0125
B.8	Acyl-CoA oxidase 2	98.4	86.8	99	99.6	0.0552	0.0046
B.9	Predicted protein 9	95.8	75.1	94.9	95.6	0.1019	0.0175
B.10	DREB3	96.8	-	97	98.4	0.1179	0.0127
C.7	SH3 domain-containing protein 2 (SH3P2)	98.7	82.6	98.9	99.4	0.0368	0.0051
C.8	MKRP2	98.5	92.2	99.3	99.7	0.0465	0.0062
C.9	Salt tolerance homolog 2	95.2	94.7	95.1	95.8	0.0586	0.0062
C.10	DHNb	96.8	85.2	97.2	98.2	0.1050	0.0099
C.11	Transcription factor/zinc-mediated transcriptional activator (SHL1)	90.7^a^					
D.2	Anthocyanidin reductase	97.8	83.7	98.8	99.1	0.0706	0.0039
D.3	Serine/threonine kinase	98.2	91.7	96.8	97.9	0.0252	0.0162

**Average**		**97.15**	**88.66**	**97.19**	**97.84**	**0.0518**	**0.0071**

^a ^Due to an unequal number of exons and introns within these homoeologues, nucleotide identity was calculated from the genomic sequence including both the exons and introns. Protein identity and similarity were not calculated.

The resulting protein average identity and similarity between the homoeologous genes was 97.19 and 97.84% respectively (Table 2). Within these amino acid sequences, the number of synonymous substitutions per synonymous site (Ks) ranged from 0 to 0.1179, while the number of non-synonymous substitutions per non-synonymous site (Ka) ranged from 0 to 0.0175. Ks values may be used to estimate an approximate time of divergence between the O and P' sub-genomes, assuming that synonymous mutations are selectively neutral and would therefore increase in a linear fashion over time. Using the median Ks value (0.05085), the predicted time of divergence was estimated as 4.2 million years ago (Mya).

When the four regions were compared, it was evident that the homoeologous genes from region A were most conserved, followed by those of regions D, C and B. Genes from region A showed the highest level of conservation across all measurements (Table 2). However, the high intron nucleotide identity was the most striking feature, being significantly higher than for the 3 other regions (p = 0.01) and when averaged (99.55%), was slightly higher than the exon nucleotide identity of the same region (99.53%).

There were no predicted genes unique to either genome within the overlap region for regions B and C, while region A contained 3 genes unique to the O sub-genome (A.8, A.9 and A.14) and 2 genes unique to the P' sub-genome (A.12 and A.13). Region D also contained a single predicted gene specific to the O sub-genome (D.5) and two genes from the P' sub-genome (D.1, D.4). While it is possible that these unique genes do have a homoeologous counterpart outside the region of overlap, none were identified in the full-length sequence.

Levels of conservation between sub-genomes across intergenic sequence was visualised through VISTA plots for each region (Figure 1). The highest level of conservation was observed from region A, where the conserved intergenic space never declined beyond 94% nucleotide identity. This observation is consistent with the higher level of nucleotide identity identified between introns of genes from both sub-genomes within this region. The only part of region A that is not highly conserved is the section containing predicted sub-genome-specific genes, in which no similarity resides. Although region A appears to display the highest levels of conservation between homoeologous genes and intergenic space, the largest number of sub-genome-specific predicted genes are also present. Regions B, C and D differ from A in that the intergenic sequence shows considerably lower conservation. The predicted homoeologous genes within these regions are conserved, as is sequence extending upstream and downstream from the coding sequence, which presumably includes promoter elements. The intergenic space, however, mostly contains large areas of complete divergence interrupted by short regions of high nucleotide identity. As with region A, similarity between sub-genomes is eliminated in those cases in which sub-genome-specific genes (region D) and transposable elements (region B) are predicted. An exception to this rule is gene D.5, which was only predicted from the O sub-genome, but appears to be partially conserved in the P' sub-genome.

### Transposable elements

A total of 36 transposable elements (TEs) were identified across all sequenced BACs (Additional file 2). Of these 36 TEs, 10 are classified as DNA transposons, four non-LTR retrotransposons, 21 LTR-retrotransposons and one is unclassified. The LTR retrotransposons were identified as belonging to the Copia (18) and Gypsy (3) classes. No TEs were identified from two BACs; wc38j22 (region A, sub-genome O) and wc36e03 (region D, sub-genome P'). From the remaining six BACs, the number of TEs predicted per BAC ranged from 1 to 12, representing 0.3 to 22.2% of the derived sequence. The average prevalence of TEs across these six BACs was one element every 19.6 kb. Of all the TEs identified, most were located in regions for which the sequence from each sub-genome did not overlap. There were however, seven elements present in areas of overlap, each being present in only one sub-genome, suggesting that these elements were inserted following divergence of the two sub-genomes.

### Microsynteny with *Medicago truncatula*, *Lotus japonicus *and *Arabidopsis thaliana*

Evaluation of microsynteny was performed between the four sequenced white clover genomic regions and the draft genomes of the model legumes *Medicago truncatula *and *Lotus japonicus*, as well as *Arabidopsis thaliana *(Table 3), and the quality of synteny was determined (Table 4).

**Table 3:** Clones identified as syntenic to *Trifolium repens *(Tr) in *Medicago truncatula *(Mt), *Lotus japonicus *(Lj) and *Arabidopsis thaliana *(At) genomes

Region	*Tr*	*Mt*		*Lj*		*At*	
	**Homoeologous group**	**Clone**	**Chr**	**Clone**	**Chr**	**Clone**	**Chr**

A.1	3			CM0050	1	T22C5	1
A.2	3			CM0096	5	MMG4	5
B	4	AC202506	4	CM0307	4	MQN23	5
C	3	AC140722 CU019606	3	CM0113	1	T22F8	4
D	4			CM1616	4		

**Table 4:** Synteny quality

Region	Reference sub-genome	**Reference length compared (kb)**^**a**^	Syntenic species	**Syntenic length compared (kb)**^**a**^	**Number of shared genes**^**a**^	**Percentage synteny quality**^**b**^
A.1	O	60	Lj	47	5	62.5
	P'	53	Lj	21	3	54.6
	O	50	At	24	3	50.0
	P'	85	At	23	3	46.2
A.2	O	48	Lj	95	4	40.0
	P'	85	Lj	113	3	27.3
	O	41	At	19	9	42.9
	P'	85	At	22	2	66.7
B	O	12	Mt	17	3	100
	P'	68	Mt	50	6	75.0
	O	33	Lj	32	6	100
	P'	69	Lj	72	9	81.8
	O	33	At	18	5	90.9
	P'	31	At	18	5	90.9
C	O	151	Mt	139	15	69.8
	P'	41	Mt	28	6	66.7
	O	81	Lj	43	8	85.7
	P'	41	Lj	28	3	85.7
	O	101	At	38	5	50
	P'	17	At	13	2	80
D	O	17	Lj	16	3	100
	P'	5	Lj	7	2	100

^a ^Calculated from the first to last shared gene in the region.

^b ^Synteny quality is calculated as twice the number of gene matches divided by the total number of genes in both segments, excluding TEs.

#### Region A

**Figure 2:**
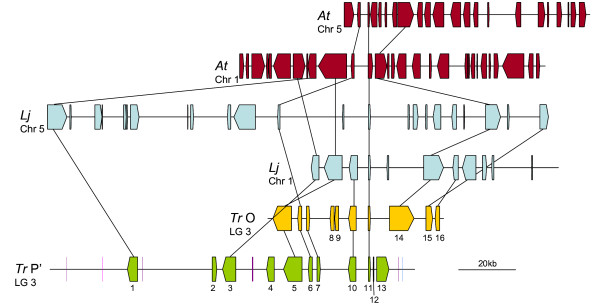
**Overview of synteny between *Trifolium repens *(*Tr*) homoeologous region A, *Lotus japonicus *(*Lj*) and *Arabidopsis thaliana *(*At*)**. Block arrows represent predicted genes from each species, the arrow direction indicating the transcriptional orientation. Gene numbering within *T. repens *corresponds to the nomenclature in Additional file 1. Coloured rectangles represent transposable elements: blue are Copia LTR retrotransposons, pink are Gypsy LTR retrotransposons and violet are DNA transposons. Identified homoeologues and orthologues of predicted white clover genes are linked by a black line.

For region A, two orthologous regions were identified on chromosomes 1 and 5 of both *L. japonicus *and *A. thaliana *(Figure 2). Synteny with *L. japonicus *is higher for the chromosome 1 region (clone CM0050) than the duplicated fragment of chromosome 5 (CM0096), with average synteny quality values of 59 and 34% respectively. Within *A. thaliana*, differences of conservation between the two clones is more difficult to distinguish based on calculated values, but based on the total number of conserved genes (6 and 3), the region on chromosome 5 (*At*5) would again appear the least conserved of the two. All genes conserved in *L. japonicus *are in the same order and orientation as found in white clover, with the exception of gene A.1 which is present on the opposite strand in *L. japonicus*. Of the six conserved genes identified in the *A. thaliana *genome, five retain the same order and orientation, while gene A.3 is present on the opposite strand.

No evidence of conservation between white clover and *M. truncatula *could be identified for region A, despite the much closer evolutionary distance between these two species as to affinities with *L. japonicus *or *A. thaliana*. A BLASTN search of the GenBank database using gene A.11 (ZPT2) returned a highly similar (e value = 0) *M. truncatula *EST (BT051747), indicating that this gene at least is present in the *M. truncatula *genome, but is not represented in the current public release genome draft.

#### Region B

**Figure 3:**
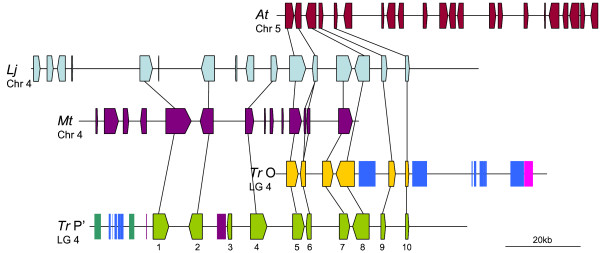
**Overview of synteny between *Trifolium repens *(*Tr*) homoeologous region B, *Lotus japonicus *(*Lj*) and *Arabidopsis thaliana *(*At*)**. Block arrows represent predicted genes from each species, the arrow direction indicating the transcriptional orientation. Gene numbering within *T. repens *corresponds to the nomenclature in Additional file 1. Coloured rectangles represent transposable elements: blue are Copia LTR retrotransposons, pink are Gypsy LTR retrotransposons, violet are DNA transposons and deep green are non-LTR retrotransposons. Identified homoeologues and orthologues of predicted white clover genes are linked by a black line.

Region B was found to be conserved in all three model species studied (Figure 3), and all orthologous genes retain the same order and orientation between the four species. The syntenic region identified within *M. truncatula *is a Phase One assembled clone consisting of 20 unordered fragments, however, only the two fragments (68,591 bp and 6,248 bp) containing the white clover gene orthologues, which were separated by 100 unattributed nucleotides (Ns), were used for comparative analysis. The synteny quality between white clover and *M. truncatula *(average 87.5%) is slightly lower than that observed for *L. japonicus *and *A. thaliana *(both average 91%), largely because the 50 kb syntenic region from *M. truncatula *contains three extra small predicted genes which are not predicted or present in white clover.

#### Region C

**Figure 4:**
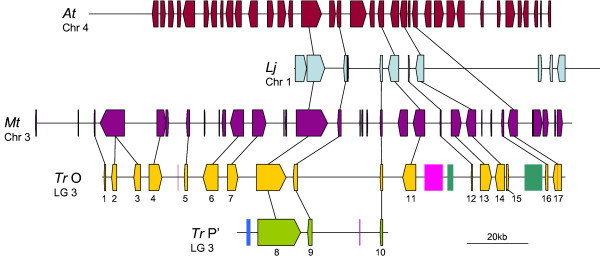
**Overview of synteny between *Trifolium repens *(*Tr*) homoeologous region C, *Lotus japonicus *(*Lj*), *Medicago truncatula *(*Mt*) and *Arabidopsis thaliana *(*At*)**. Block arrows represent predicted genes from each species, the arrow direction indicating the transcriptional orientation. Gene numbering within *T. repens *corresponds to the nomenclature in Additional file 1. Coloured rectangles represent transposable elements: blue are Copia LTR retrotransposons, pink are Gypsy LTR retrotransposons, violet are DNA transposons and deep green are non-LTR retrotransposons. Identified homoeologues and orthologues of predicted white clover genes are linked by a black line.

Overlapping clones AC140122 and CU019606 from *M. truncatula *chromosome 3 show extensive synteny with white clover region C (Figure 4). Gene C.15 is the only predicted gene absent from this conserved region, of which the basis of the prediction was identification of a white clover EST, rather than *in silico *analysis. As it is possible that gene prediction algorithms overlooked this coding region in *M. truncatula*, the level of conservation between gene C.15 and the orthologous clones was examined but failed to identify conservation, suggesting that this gene is indeed specific to white clover. Again, the average synteny quality value for this region (68%) does not accurately reflect the high level of gene retention, largely because the aligned 139 kb segment from *M. truncatula *contains 12 additional and much smaller genes that were not predicted in white clover. Comparative analysis of the two regions revealed that 11 of the genes are not conserved in the white clover genome. The *M. truncatula *gene CU019606_5, however, showed similarity (77.88% nucleotide identity) with white clover in the expected genomic location. Closer inspection revealed that this conserved area does not contain the first 120 bp of the gene CU019606_5, explaining the failure of prediction.

A phase 1 assembled *L. japonicus *clone (CM0113) which contains five islands of genic sequence, is conserved with region C. The islands of sequence in CM0113 are separated by gaps represented by 100 unattributed nucleotides (Ns), which makes comparative analysis between the two species difficult. As the order of the gene islands from the downloaded CM0113 sequence did not match that of *M. truncatula *and white clover, they were rearranged for the purposes of alignment (Figure 4). Although it is possible and probable that the six genes are conserved in order and orientation, this conjecture must be treated with caution. Nevertheless, the degree of synteny calculated between the genomes of white clover and *L. japonicus *for this region averages 86%. White clover region C is also partially conserved on *A. thaliana *chromosome 4, with a total of five genes found in the same order and orientation and an average synteny quality calculated at 65%.

#### Region D

**Figure 5:**
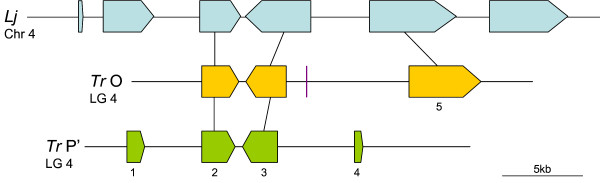
**Overview of synteny between *Trifolium repens *(*Tr*) homoeologous region D and *Lotus japonicus *(*Lj*)**. Block arrows represent predicted genes from each species, the arrow direction indicating the transcriptional orientation. Gene numbering within *T. repens *corresponds to the nomenclature in Additional file 1. The violet coloured rectangle represents a DNA transposon. Identified homoeologues and orthologues of predicted *T. repens *genes are linked by a black line.

Of the three model species studied, conservation of region D was only observed for *L. japonicus*, in which a total of three genes are conserved in both order and orientation to produce an average synteny quality of 100% (Figure 5). No similarity with the *M. truncatula *genome was observed for any of the predicted genes from region D when the version 3 genome release was searched, despite the presence of a well-characterised *ANR *(gene D.2) orthologue (e value = 0) in GenBank (AY184243).

### Conservation of orthologous genes

Similarity between white clover genes and their predicted orthologues was calculated as a measure of percent nucleotide identity between the coding regions (Additional file 3). As expected, due to the similarity between the O and P' sub-genomes, there is little difference between the nucleotide identity of genes from either of the sub-genomes and the orthologues, neither sub-genome being consistently more similar to that of either model species. When data was combined from both of the sub-genomes, the average nucleotide identity values observed with *M. truncatula*, *L. japonicus *and *A. thaliana *coding regions were 82.34, 73.48 and 56.19%, respectively. Ks values were also calculated for orthologues of the homoeologous genes, and used to estimate pair-wise times of divergence (Additional file 4). Taking the median Ks value, the divergence time from *M. truncatula *was calculated as 27.5 Mya for the O sub-genome and 26.0 Mya for the P' sub-genome. The time of divergence from *L. japonicus *was estimated at 68.0 Mya for the O sub-genome and 68.8 Mya for the P' sub-genome. These values provide an internal calibration for estimates of divergence between the O and P' sub-genomes. No sequence conservation for intergenic spaces was observed between white clover and any of the three model species.

## Discussion

Analysis of the four homoeologous BAC pairs through sequencing in this study provide an insight into white clover genome structure. The gene density observed (one gene per 9.7 kb) is lower than that of *M. truncatula *and *L. japonicus*, which has been reported as one gene per 7.94 and 5.75 kb, respectively [38]. As white clover has an estimated genome size of 956 Mb [39], which equates to two diploid sub-genomes of similar size to those of both *M. truncatula *(466 Mb) and *L. japonicus *(466 Mb) [39], observed differences in gene density are probably due to the relatively small size of the regions sampled, which has resulted in a lower observed gene density compared to the remainder of the genome. Comparisons of gene density between these three species are also potentially affected by differing methods of gene prediction used for each genome. Gene density is also lower than the range reported from the more distantly related Phaseoloid legume soybean (*Glycine max *L. Merr.) (one gene per 5.8-9.5 kb) [8,40] and that calculated from similar studies in another dicotyledonous species, cotton (*Gossypium hirsutum*) (one gene per 7.5 kb) [9]. The value is also lower than that of *A. thaliana *(one gene per 5 kb) [41], which has a much smaller genome size (157 Mb).

By selecting four homoeologous BAC pairs, this study was able to compare sub-genome-specific sequence conservation across multiple regions of the genome, and has revealed different patterns of conservation and divergence. Region A represents simultaneously both the most highly and least conserved region, the abrupt divergence between the sub-genomes being likely to be due to a small scale deletion. From the point of divergence onwards, the O sub-genome is more similar to the equivalent regions in *L. japonicus *and *A. thaliana *than to the P' sub-genome, suggesting that the deletion has occurred in the latter. Large-scale deletions have been identified as one of the few processes known to contribute to genome contraction, and occur through mechanisms such as unequal homologous recombination and illegitimate recombination [42,43], evidence for which is suggested by the presence of a short (32 bp) sequence repeat at the site of the deletion event.

The remaining three regions (B, C and D) have mostly retained homoeologous genes, although the intergenic space is much more diverged than in region A, with only abrupt short stretches of conservation. Such patterns are more typical of relationships between sub-genomes in polyploids with varying ages of synthesis [8,44-46], such that divergence times as recent as 0.5-1 Mya show a lack of conservation between homoeologous genes [47]. In contrast, a comparison of two homoeologous regions in the genome of the cotton species *G. hirsutum*, for which the sub-genomes are predicted to have diverged 5-10 Mya [48], revealed considerable conservation of intergenic regions, similar to that observed in region A of the present work. As the same evolutionary forces and selection mechanisms are not acting ubiquitously throughout the genome, it is feasible that the regions of intergenic conservation may correspond to conserved functional DNA elements, possibly involved in regulation of transcription. The major differences between the homoeologous A and D sub-genomes of *G. hirsutum *are the presence of sub-genome-specific TEs, which are believed to be responsible for the c. two-fold difference in sub-genome size [9,10]. These findings are consistent with other generic genome structure comparisons, in which transposable element proliferation is considered the main contributing factor to differences in genome size [44,49]. For the four homoeologous regions studied here, TEs did not contribute substantially to sub-genome variation, perhaps due to regional sampling bias, but of the seven TEs identified in overlap regions, all were sub-genome-specific, similar to other reports of differential accumulation [50].

As the four sampled homoeologous regions have displayed varying levels of conservation in both genic and intergenic space, it is apparent that multiple sections of the genome need to be analysed in order to draw conclusions on the divergence of resident sub-genomes within allopolyploid species. Obviously, selection and analysis of alternative genomic regions may yield different conservation patterns, and therefore extrapolation of the results from this work to the O and P' sub-genomes as a whole is limited by the data from,these four selected regions. A complete picture of sub-genome divergence, however, can only be obtained from sequencing of each sub-genome. This study is therefore significant for development of sequencing strategies, as measures of homoeologous conservation on this scale have previously not been reported for white clover.

By calculating the rate of synonymous substitutions between homoeologous genes within white clover, this study is able to estimate the time of divergence between the O and P' genomic regions selected for this study at 4.2 Mya. While this method has previously been used to determine the elapsed time since evolutionary duplication events within various species [27,28], information from large EST databases was used, rather than the smaller number of homoeologous genes used here. As a consequence, use of thjs data to estimate time since species divergence must be treated with caution. The divergence time estimated between white clover and *M. truncatula *(26.7 Mya) does, however, fall within the estimate range of Lavin et al [15], who predicted divergence between the Trifolieae and Fabeae tribes at c. 17.1 - 30.2 Mya from Bayesian analysis of two chloroplast DNA genes and a fossil-calibrated approach. The estimated figure for O and P' sub-genome separation (4.2 Mya) is also well below the 17.1 Mya minimum, as would be expected. The divergence estimate between white clover and *L. japonicus *observed in the current study (68.4 Mya) is, however, somewhat higher than the range of 47.7 - 52.7 Mya predicted by Lavin et al [15]. This is likely to be a consequence of small sampling size and unusually high Ks values for a number of these genes, as reflected in the larger standard deviation of Ks values observed for *L. japonicus *(0.6020) compared to *M. truncatula *(0.1137).

In each instance, orthologous regions identified from *M. truncatula *and *L. japonicus *were located on the chromosomes predicted from previous studies of macrosynteny [51,52]. For the two conserved regions detected from *L. japonicus *and *A. thaliana *for region A, both macrosynteny studies and observed synteny quality suggest that the fragments from chromosome 1 (region A.1) are the true orthologues and that the conserved fragments from chromosome 5 (region A.2) are duplicated regions that have been previously identified within the genome of each species [53,54]. The relative lack of utility of the *M. truncatula *genome for identification of microsyntenic regions was unexpected and surprising. As the two species are located within the same sub-family of Galegoid legumes, the *M. truncatula *genome sequence is the most closely related available reference genome for white clover. However, *M. truncatula *orthologous regions were only identified for two of the four sequenced white clover regions. It is possible that due to sampling of only two white clover chromosomes, (Table 1) this study has obtained a biased assessment of utility of the *M. truncatula *genome release. For the two regions in which white clover-*M. truncatula *microsynteny was observed, the quality level suggests that the two genomes are similar in structure, as expected from phylogenetic and macrosynteny studies [15,52], and observations in this study are due to identification of gaps in the current genome draft release. This is further supported by the identification of *M. truncatula *orthologues for the genes A.11 (*ZPT2*) and D.2 (*ANR*) in GenBank which are absent from the *M. truncatula *genome release 3.0. As this *M. truncatula *genome release is preliminary and incomplete, it is likely that future genome releases will reveal syntenic clones for white clover regions A and D.

In contrast, despite a greater evolutionary distance, *L. japonicus *genome comparison have obtained impressive levels of conservation (average 84% synteny quality) for all corresponding white clover regions. As the two species are predicted to have diverged approximately 50 Mya, this level of microsynteny is impressive and larger than that previously observed for comparisons between other legume species. Mudge et al. [40] reported an average synteny quality of 60% between 400 kb of sequence from *M. truncatula *and *G. max*, which are estimated to have diverged approximately 54 Mya [15], and similar levels have also been observed between *M. truncatula *and *L. japonicus *(62%) [38,51]. While each individual region analysed here is smaller than in previous studies, and hence may once again be vulnerable to biased sampling, the high level of conservation is consistent across four separate areas of the genome. Conservation with the *A. thaliana *genome was also higher than might have been expected, given that the two species are thought to have shared a common ancestor approximately 100 Mya [55,56]. Conserved regions were identified for three of the four regions analysed, and synteny quality averaged 68%. This level of conservation is consistent with other legume-specific studies, in which blocks of synteny in *A.thaliana *have been found for *M. truncatula*, *L. japonicus *and *G. max *[40,57,58].

Evaluation of microsynteny in this study has important implications for the design of whole genome sequencing strategies. The fully sequenced genomes of model species provide fundamental resources for translational genomics, and can potentially act as scaffolds for both physical mapping and next-generation whole genome sequencing of closely related species. The *Oryza *Map Alignment Project (OMAP) provides an example of this process, such that physical maps of an additional 12 *Oryza *species are being generated through the use of the fully sequenced rice genome as a framework for construction [59]. With the growth of next-generation sequencing technologies, production of a draft white clover genome sequence is a realistic possibility. The similarity between the O and P' sub-genomes as observed here, however, would render an accurate sequence assembly of the allotetraploid genome extremely difficult. The most effective strategy, therefore, would probably be to first sequence the genome of *T. occidentale*, the most probable of the diploid progenitors. Based on this study, it is likely that the genome drafts of both *M. truncatula *and *L. japonicus *would then be able to act as scaffolds for the whole genome sequence assembly. While *de novo *assembly may be challenging from the short reads generated by the next-generation sequencing platforms, a strategy combining single and paired-end reads (with c. 2-5 kb inserts) is conceivable based upon the gene density and sub-genome sequence conservation described in this study. The most successful genome assembly, however, is likely to be the result of a hybrid *de novo *and reference physical mapping approaches.

## Conclusions

This work provides the first detailed analysis of homoeologous sub-genome similarity across selected white clover genomic regions in *T. repens*. A high level of homoeologous gene retention was generally observed for all four genomic regions studied, except in those cases in which small scale deletions were present. The degree of intergenic sequence divergence, however, varied between each region. This research also detected conserved microsynteny between *T. repens *and *L. japonicus *for each of the analysed regions. Surprisingly, microsynteny between *T. repens *and *M. truncatula *was detected for only two of the four regions, but the quality of synteny was high. In most cases, conserved gene order could also be extended to *A. thaliana*. These findings suggest that the sequenced genomes of the model legumes, and in particular that of *L. japonicus*, will be valuable for the future development of genomic resources in white clover.

## Authors' contributions

MH selected, sequenced, assembled and annotated the BACs, performed the sequence analysis and drafted the manuscript. NC and JF co-conceptualised and coordinated the project, contributed to data interpretation and assisted in drafting the manuscript. TS contributed to data interpretation and assisted in drafting the manuscript. GS co-conceptualised the project and assisted in drafting the manuscript. All authors read and approved the final manuscript.

## Supplementary Material

Additional file 1 **Predicted gene features of four homoeologous regions in white clover**. List of all genes predicted across each homoeologous region and details of their exon and intron lengths. Click here for file

Additional file 2 **Transposable elements identified in each white clover homoeologous region**. List of all transposable elements identified within the four homoeologous regions studied and details of their class and size. Click here for file

Additional file 3 **Nucleotide identity between genes of white clover and model species**. List of genes from *Medicago truncatula *(Mt), *Lotus japonicus *(Lj) and *Arabidopsis thaliana *(At) identified as orthologous to the predicted white clover genes and the percent nucleotide identity between each pair. Click here for file

Additional file 4 **Calculated Ks values**. Table listing the number of synonymous substitutions per synonymous site (Ks) between predicted white clover genes and their orthologues from *Medicago truncatula *(Mt), *Lotus japonicus *(Lj) and *Arabidopsis thaliana *(At). Click here for file
