# Supplementary material for: Comparison of homoeolocus organisation in paired BAC clones from white clover (Trifolium repens L.) and microcolinearity with model legume species
Source: BMC Plant Biol. 2010 May 24;10:94. doi: 10.1186/1471-2229-10-94 (PMC3095360; doi:10.1186/1471-2229-10-94)
Supplement: Additional file 1 — Predicted gene features of four homoeologous regions in white clover. List of all genes predicted across each homoeologous region and details of their exon and intron lengths. [file 1471-2229-10-94-S1.DOC]

### Additional file 1 - Predicted gene features of four homoeologous regions in white clover

| **Gene identity** | **Putative function^a^** | **Sub-genome attribution of supporting EST** | **BAC identity** | | **Total exon length (bp)** | | **Total intron length (bp)** | | **No. exons^b^** | | **Average exon length (bp)** | | **Average intron length (bp)** | |
| --- | --- | --- | --- | --- | --- | --- | --- | --- | --- | --- | --- | --- | --- | --- |
|  |  |  | O | P’ | O | P’ | O | P’ | O | P’ | O | P’ | O | P’ |
| A.1 | Predicted protein 1 |  | - | wc11l07 | - | 1308 | - | 2248 | - | 12 | - | 109 | - | 204 |
| A.2 | Cyclin d |  | - | wc11l07 | - | 951 | - | 640 | - | 5 | - | 190 | - | 160 |
| A.3 | Calcium ATPase |  | - | wc11l07 | - | 3060 | - | 1576 | - | 7 | - | 437 | - | 263 |
| A.4 | E3 ubiquitin ligase |  | - | wc11l07 | - | 1017 | - | 1601 | - | 2 | - | 509 | - | 1601 |
| A.5 | Predicted protein 2 |  | wc38j22 | wc11l07 | 2667 | 2673 | 3811 | 3809 | 9 | 9 | 296 | 297 | 476 | 476 |
| A.6 | Predicted protein 3 |  | wc38j22 | wc11l07 | 873 | 873 | 461 | 461 | 5 | 5 | 175 | 175 | 115 | 115 |
| A.7 | Galactose oxidase |  | wc38j22 | wc11l07 | 1062 | 1062 | 71 | 72 | 2 | 2 | 531 | 531 | 71 | 72 |
| A.8 | Unknown protein | O | wc38j22 | - | 609 | - | 808 | - | 6 | - | 102 | - | 162 | - |
| A.9 | ATPase | O | wc38j22 | - | 561 | - | 792 | - | 3 | - | 187 | - | 396 | - |
| A.10 | Adeninephosphoribosyl transferase |  | wc38j22 | wc11l07 | 561 | 561 | 2093 | 2093 | 6 | 6 | 94 | 94 | 419 | 419 |
| A.11 | ZPT2 |  | wc38j22 | wc11l07 | 720 | 732 | 0 | 0 | 1 | 1 | 720 | 732 | - | - |
| A.12 | Cysteine protease |  | - | wc11l07 | - | 219 | - | 0 | - | 1 | - | 219 | - | - |
| A.13 | Predicted protein 4 |  | - | wc11l07 | - | 2616 | - | 1703 | - | 4 | - | 654 | - | 568 |
| A.14 | Transcription initiation factor |  | wc38j22 | - | 2805 | - | 6455 | - | 13 | - | 216 | - | 538 | - |
| A.15 | Proteinase inhibitor |  | wc38j22 | - | 2304 | - | 0 | - | 1 | - | 2304 | - | - | - |
| A.16 | Predicted protein 5 |  | wc38j22 | - | 987 | - | 614 | - | 3 | - | 329 | - | 307 | - |
| B.1 | Predicted protein 6 |  | - | wc88n22 | - | 1515 | - | 2675 | - | 8 | - | 189 | - | 382 |
| B.2 | Myo-inositol-1-phosphate synthase | P’ | - | wc88n22 | - | 1536 | - | 2062 | - | 9 | - | 171 | - | 258 |
| B.3 | Predicted protein 7 |  | - | wc88n22 | - | 1056 | - | 111 | - | 2 | - | 528 | - | 111 |
| B.4 | Predicted protein 8 |  | - | wc88n22 | - | 990 | - | 3476 | - | 7 | - | 141 | - | 579 |
| B.5 | Bristled 1 |  | wc113f04 | wc88n22 | 1749 | 1557 | 1363 | 1543 | 10 | 10 | 175 | 156 | 151 | 171 |
| B.6 | Ethylene insensitive 3 |  | wc113f04 | wc88n22 | 1257 | 1365 | 97 | 0 | 2 | 1 | 629 | 1365 | 97 | - |
| B.7 | bZIP transcription factor |  | wc113f04 | wc88n22 | 1992 | 1992 | 824 | 761 | 5 | 5 | 398 | 398 | 206 | 190 |
| B.8 | Acyl-CoA oxidase 2 |  | wc113f04 | wc88n22 | 2058 | 2058 | 2833 | 2337 | 7 | 7 | 294 | 294 | 472 | 390 |
| B.9 | Predicted protein 9 |  | wc113f04 | wc88n22 | 876 | 885 | 881 | 1495 | 4 | 4 | 219 | 221 | 294 | 498 |
| B.10 | DREB3 | O | wc113f04 | wc88n22 | 915 | 915 | 0 | 0 | 1 | 1 | 915 | 915 | - | - |
| C.1 | Predicted protein 10 |  | wc99k01 | - | 282 | - | 245 | - | 2 | - | 141 | - | 245 | - |
| C.2 | Zinc knuckle (CCHC-type) family protein |  | wc99k01 | - | 771 | - | 856 | - | 3 | - | 257 | - | 428 | - |
| C.3 | Zinc knuckle (CCHC-type) family protein | O | wc99k01 | - | 825 | - | 1307 | - | 3 | - | 275 | - | 654 | - |
| C.4 | Metal ion binding |  | wc99k01 | - | 771 | - | 3463 | - | 9 | - | 86 | - | 433 | - |
| C.5 | Zinc finger (GATA type) family protein | O | wc99k01 | - | 939 | - | 67 | - | 2 | - | 470 | - | 67 | - |
| C.6 | Ferredoxin hydrogenase | O | wc99k01 | - | 1488 | - | 3471 | - | 12 | - | 124 | - | 316 | - |
| C.7 | SH3 domain-containing protein 2 (SH3P2) | O | wc99k01 | wc32k23 | 1092 | 1092 | 2334 | 1797 | 10 | 10 | 109 | 109 | 259 | 200 |
| C.8 | MKRP2 |  | wc99k01 | wc32k23 | 3096 | 3216 | 6649 | 6230 | 24 | 24 | 129 | 134 | 289 | 271 |
| C.9 | Salt tolerance homolog 2 |  | wc99k01 | wc32k23 | 825 | 852 | 529 | 544 | 3 | 3 | 275 | 284 | 265 | 272 |
| C.10 | DHNb | O+P’ | wc99k01 | wc32k23 | 657 | 654 | 223 | 237 | 2 | 2 | 329 | 327 | 223 | 237 |
| C.11 | Transcription factor/ zinc-mediated transcriptional activator (SHL1) | P’ | wc99k01 | wc32k23 | 879 | 822 | 3327 | 3466 | 8 | 7 | 110 | 117 | 475 | 578 |
| C.12 | Predicted protein 11 |  | wc99k01 | - | 282 | - | 82 | - | 2 | - | 141 | - | 82 | - |
| C.13 | 26S proteasome AAA-ATPase subunit RPT4a |  | wc99k01 | - | 1320 | - | 2297 | - | 11 | - | 120 | - | 230 | - |
| C.14 | Inositol monophosphatase |  | wc99k01 | - | 891 | - | 2011 | - | 8 | - | 111 | - | 287 | - |
| C.15 | Unknown protein |  | wc99k01 | - | 529 | - | 210 | - | 2 | - | 265 | - | 210 | - |
| C.16 | Dehydrin | O | wc99k01 | - | 552 | - | 279 | - | 2 | - | 276 | - | 279 | - |
| C.17 | Protein/zinc ion binding |  | wc99k01 | - | 1287 | - | 146 | - | 3 | - | 429 | - | 73 | - |
| D.1 | F-box family protein |  | - | wc36e03 | - | 1113 | - | 0 |  | 1 | - | 1113 | - | - |
| D.2 | Anthocyanidin reductase | P’ | wc88b23 | wc36e03 | 1017 | 1020 | 1293 | 1064 | 6 | 6 | 170 | 170 | 259 | 213 |
| D.3 | Serine/threonine kinase |  | wc88b23 | wc36e03 | 1128 | 1128 | 1257 | 1053 | 7 | 7 | 161 | 161 | 210 | 176 |
| D.4 | Predicted protein 12 |  | - | wc36e03 | - | 411 | - | 103 |  | 2 | - | 206 | - | 103 |
| D.5 | Translation initiation factor-related |  | wc88b23 | - | 1854 | - | 2643 | - | 6 | - | 309 | - | 529 | - |
| **Average** |  |  |  |  | **1180** | **1308** | **1630** | **1726** | **6** | **6** | **330** | **365** | **288** | **340** |

^a^ ‘Predicted protein’ indicates that the predicted gene shows no similarity to any previously functionally characterized gene in GenBank. ‘Unknown protein’ indicates that the gene was predicted solely based on alignment of an EST.

^b^ Number of introns per gene is n-1 where n is the number of exons.
