# Supplementary material for: Comparison of homoeolocus organisation in paired BAC clones from white clover (Trifolium repens L.) and microcolinearity with model legume species
Source: BMC Plant Biol. 2010 May 24;10:94. doi: 10.1186/1471-2229-10-94 (PMC3095360; doi:10.1186/1471-2229-10-94)
Supplement: Additional file 2 — Transposable elements identified in each white clover homoeologous region. List of all transposable elements identified within the four homoeologous regions studied and details of their class and size. [file 1471-2229-10-94-S2.DOC]

### Additional file 2 - Transposable elements identified in each white clover homoeologous region

| Class | Region | Size (bp) | | In overlap region? |
| --- | --- | --- | --- | --- |
|  |  | O sub-genome | P’ sub-genome |  |
| DNA transposon | A | - | 63 | no |
| Gypsy | A | - | 68 | no |
| DNA transposon | A | - | 73 | no |
| DNA transposon | A | - | 335 | no |
| DNA transposon | A | - | 64 | yes |
| Copia | A | - | 91 | yes |
| Non-LTR retrotransposon | B | - | 1712 | no |
| Copia | B | - | 507 | no |
| Non-LTR retrotransposon | B | - | 1377 | no |
| DNA transposon | B | - | 2301 | no |
| Copia | B | - | 79 | no |
| Copia | B | - | 541 | no |
| Copia | B | - | 610 | no |
| Copia | B | - | 341 | no |
| Copia | B | - | 362 | no |
| DNA transposon | B | - | 138 | no |
| Copia | B | 4397 | - | yes |
| Copia | B | 3945 | - | yes |
| Unclassified | B | 3636 | - | no |
| Gypsy | B | 2306 | - | no |
| Copia | B | 75 | - | no |
| Copia | B | 95 | - | no |
| Copia | B | 73 | - | no |
| Copia | B | 906 | - | no |
| Copia | B | 164 | - | no |
| Copia | B | 356 | - | no |
| Copia | B | 70 | - | no |
| Copia | B | 213 | - | no |
| DNA transposon | C | 49 | - | no |
| Non-LTR retrotransposon | C | 5777 | - | no |
| Non-LTR retrotransposon | C | 1875 | - | no |
| Gypsy | C | 5972 | - | no |
| DNA transposon | C | 64 | - | no |
| DNA transposon | C | - | 248 | yes |
| Copia | C | - | 1242 | yes |
| DNA transposon | D | 67 | - | yes |
