# Supplementary material for: Comparison of homoeolocus organisation in paired BAC clones from white clover (Trifolium repens L.) and microcolinearity with model legume species
Source: BMC Plant Biol. 2010 May 24;10:94. doi: 10.1186/1471-2229-10-94 (PMC3095360; doi:10.1186/1471-2229-10-94)
Supplement: Additional file 3 — Nucleotide identity between genes of white clover and model species. List of genes from Medicago truncatula (Mt), Lotus japonicus (Lj) and Arabidopsis thaliana (At) identified as orthologous to the predicted white clover genes and the percent nucleotide identity between each pair. [file 1471-2229-10-94-S3.DOC]

### Additional file 3 - Nucleotide identity between genes of white clover and model species

| **Gene identity** | **Putative function** | ***Mt* orthologue** | **Nucleotide identity (%)** | | ***Lj* orthologue** | **Nucleotide identity (%)** | | ***At* orthologue** | **Nucleotide identity (%)** | |
| --- | --- | --- | --- | --- | --- | --- | --- | --- | --- | --- |
|  |  |  | O | P’ |  | O | P’ |  | O | P’ |
| A.1 | Predicted protein 1 | - | - | - | CM0096.650 | - | 72.4 | AT1G27760 | - | 58.8 |
| A.3 | Calcium ATPase | - | - | - | CM0050.10 | - | 89.0 | AT1G27770 | - | 74.5 |
| A.5 | Predicted protein 2 | - | - | - | CM0050.20 | 78.7 | 79.0 | AT1G27752 | 57.8 | 57.2 |
| A.6 | Predicted protein 3 | - | - | - | CM0096.500 | 74.1 | 74.1 | AT1G27740  AT5G43175 | 56.2  51.9 | 55.4  51.2 |
| A.10 | Adeninephosphoribosyl transferase | - | - | - | CM0050.40 | 83.8 | 83.8 | - | - | - |
| A.11 | ZPT2 | - | - | - | CM0050.50  CM0096.480 | 69.4  61.0 | 68.0  61.9 | AT1G27730  AT5G43170 | 53.4  53.6 | 50.8  51.6 |
| A.14 | Transcription initiation factor | - | - | - | CM0050.80  CM0096.390 | 76.2  70.6 | -  - | AT1G27720  AT5G43130 | 54.4  49.2 | -  - |
| A.15 | Proteinase inhibitor | - | - | - | CM0096.370 | 76.4- | - | - | - | - |
| A.16 | Predicted protein 5 | - | - | - | CM0050.90 | 84.2 | - | - | - | - |
| B.1 | Predicted protein 6 | Medtr4g140350 | - | 88.1 | CM0307.70 | - | 81.1 | - | - | - |
| B.2 | Myo-inositol-1-phosphate synthase | Medtr4g140360 | - | 93.8 | CM0307.90 | - | 87.6 | - | - | - |
| B.4 | Predicted protein 8 | Medtr4g140370 | - | 83.8 | CM0307.120 | - | 83.5 | - | - | - |
| B.5 | Bristled 1 | Medtr4g140410 | 90.3 | 78.5 | CM0307.130 | 83.5 | 73.3 | AT5G65090 | 65.5 | 58.1 |
| B.6 | Ethylene insensitive 3 | Medtr4g140420 | 81.8 | 79.1 | CM0307.140 | 64.7 | 67.7 | AT5G65100 | 48.2 | 52.7 |
| B.7 | bZIP transcription factor | Medtr4g140290 | 86.3 | 86.5 | CM0307.150 | 75.9 | 75.8 | - | - | - |
| B.8 | Acyl-CoA oxidase 2 | - | - | - | CM0307.160 | 86.0 | 85.7 | AT5G65110 | 74.2 | 73.6 |
| B.9 | Predicted protein 9 | - | - | - | CM0307.170 | 63.1 | 62.3 | AT5G65120 | 46.6 | 47.3 |
| B.10 | DREB3 | - | - | - | CM0307.180 | 64.8 | 65.1 | AT5G65130 | 56.0 | 54.9 |
| C.1 | Predicted protein 10 | Medtr3g163000 | 78.3 | - | - | - | - | - | - | - |
| C.2 | Zinc knuckle (CCHC-type) family protein | Medtr3g162980 | 80.6 | - | - | - | - | - | - | - |
| C.3 | Zinc knuckle (CCHC-type) family protein | Medtr3g162990 | 87.6 | - | - | - | - | - | - | - |
| C.4 | Metal ion binding | Medtr3g162970 | 72.2 | - | - | - | - | - | - | - |
| C.5 | Zinc finger (GATA type) family protein | Medtr3g162960 | 83.7 | - | - | - | - | - | - | - |
| C.6 | Ferredoxin hydrogenase | Medtr3g162950 | 91.2 | - | - | - | - | - | - | - |
| C.7 | SH3 domain-containing protein 2 (SH3P2) | Medtr3g162940 | 92.1 | - | - | - | - | - | - | - |
| C.8 | MKRP2 | Medtr3g162930 | 91.9 | 92.6 | CM0113.20 | 86.4 | 85.0 | AT4G39050 | 68.3 | 68.6 |
| C.9 | Salt tolerance homolog 2 | Medtr3g162910 | 84.3 | 42.0 | CM0113.30 | 74.4 | 41.9 | AT4G39070 | 52.8 | 36.6 |
| C.10 | DHNb | Medtr3g162880 | 82.5 | 82.8 | CM0113.70 | 65.4 | 66.0 | - | - | - |
| C.11 | Transcription factor/ zinc-mediated transcriptional activator (SHL1) | Medtr3g162860 | 69.9 | - | CM0113.60 | 61.5 | - | AT4G39100 | 53.9 | - |
| C.12 | Predicted protein 11 | Medtr3g162830 | 91.8 | - | CM0113.50 | 44.6 | - | - | - | - |
| C.13 | 26S proteasome AAA-ATPase subunit RPT4a | Medtr3g162820 | 81.6 | - | - | - | - | - | - | - |
| C.14 | Inositol monophosphatase | Medtr3g162810 | 82.8 | - | CM0113.40 | 80.0 | - | AT4G39120 | 60.7 | - |
| C.16 | Dehydrin | Medtr3g162790 | 81.4 | - | - | - | - | - | - | - |
| C.17 | Protein/zinc ion binding | Medtr3g162780 | 83.4 | - | CM0113.150 | 75.2 | - | AT4G39140 | 55.5 | - |
| D.2 | Anthocyanidin reductase | - | - | - | CM1616.360 | 85.3 | 84.9 | - | - | - |
| D.3 | Serine/threonine kinase | - | - | - | CM1616.370 | 69.3 | 69.7 | - | - | - |
| D.4 | Predicted protein 12 | - | - | - | CM1616.380 | 78.2 | - | - | - | - |
