# Supplementary material for: Comparison of homoeolocus organisation in paired BAC clones from white clover (Trifolium repens L.) and microcolinearity with model legume species
Source: BMC Plant Biol. 2010 May 24;10:94. doi: 10.1186/1471-2229-10-94 (PMC3095360; doi:10.1186/1471-2229-10-94)
Supplement: Additional file 4 — Calculated Ks values. Table listing the number of synonymous substitutions per synonymous site (Ks) between predicted white clover genes and their orthologues from Medicago truncatula (Mt), Lotus japonicus (Lj) and Arabidopsis thaliana (At). [file 1471-2229-10-94-S4.DOC]

### Additional file 1 - Calculated Ks values

| **Gene ID** | **Putative function** | **Mt orthologue** | **Ks** | **Ks** | **Lj orthologue** | **Ks** | **Ks** |
| --- | --- | --- | --- | --- | --- | --- | --- |
|  |  |  | O | P’ |  | O | P’ |
| A.5 | Predicted protein 2 | - | - | - | CM0050.20 | 0.4403 | 0.4410 |
| A.6 | Predicted protein 3 | - | - | - | CM0096.500 | 0.7272 | 0.7272 |
| A.10 | Adeninephosphoribosyl transferase | - | - | - | CM0050.40 | 0.5244 | 0.5244 |
| A.11 | ZPT2 | - | - | - | CM0050.50  CM0096.480 | 0.9332  1.8069 | 1.0197  1.8358 |
| B.5 | Bristled 1 | Medtr4g140410 | 0.3642 | 0.3458 | CM0307.130 | 0.6766 | 0.6915 |
| B.6 | Ethylene insensitive 3 | Medtr4g140420 | 0.3396 | 0.3622 | CM0307.140 | 1.005 | 0.9834 |
| B.7 | bZIP transcription factor | Medtr4g140290 | 0.2718 | 0.2663 | CM0307.150 | 0.4223 | 0.4315 |
| B.8 | Acyl-CoA oxidase 2 | Medtr4g140300 | -^a^ | -^a^ | CM0307.160 | 0.4871 | 0.5110 |
| B.9 | Predicted protein 9 | - | - | - | CM0307.170 | 0.9518 | 0.9510 |
| B.10 | DREB3 | - | - | - | CM0307.180 | 0.9803 | 0.9863 |
| C.7 | SH3 domain-containing protein 2 (SH3P2) | Medtr3g162940 | 0.1802 | 0.1529 | - | - | - |
| C.8 | MKRP2 | Medtr3g162930 | 0.2723 | 0.2535 | CM0113.20 | -^a^ | -^a^ |
| C.9 | Salt tolerance homolog 2 | Medtr3g162910 | 0.3312 | 0.2881 | CM0113.30 | 1.1269 | 1.0295 |
| C.10 | DHNb | Medtr3g162880 | 0.4084 | 0.4433 | CM0113.70 | 2.8401 | 2.4695 |
| C.11 | Transcription factor/ zinc-mediated transcriptional activator (SHL1) | Medtr3g162860 | 0.5141 | 0.5675 | CM0113.60 | 1.1420 | 1.3729 |
| D.2 | Anthocyanidin reductase | - | - | - | CM1616.360 | 0.5628 | 0.5832 |
| D.3 | Serine/threonine kinase | - | - | - | CM1616.370 | 0.4844 | 0.4629 |
| **Median Ks** |  |  | 0.3354 | 0.3170 |  | 0.8302 | 0.8391 |
| **Ks Std dev** |  |  | 0.1002 | 0.1273 |  | 0.6669 | 0.5371 |
| **Divergence time (Mya)** |  |  | 27.5 | 26.0 |  | 68.0 | 68.8 |
